# Accumulation of embryos over 3 natural modified IVF (ICSI) cycles followed by transfer to improve the outcome of poor responders

**Published:** 2019-03

**Authors:** AK Datta, S Campbell, N Felix, G Nargund

**Affiliations:** Create Fertility, 150, Cheapside, St Paul’s London EC2 V6ET, United Kingdom

**Keywords:** IVF (ICSI), Natural modified IVF, poor responders, multiple IVF-cycles, accumulation of embryos, cumulative outcomes

## Abstract

**Background:**

Alternatives to improve treatment outcomes in poor responders are needed. For this we studied whether multiple (x3) Natural Modified (NM)-IVF(ICSI) cycles followed by an embryo transfer (ET) from the accumulated embryos can improve the treatment outcomes in poor responders.

**Method:**

A retrospective analysis was applied to a pool of participants qualifying as poor responders according to the Bologna criteria. This was performed over a 2-year IVF center database with a Study Group including women with a minimum of 3 cycles of NM-IVF (ICSI) and subsequent vitrified-thawed ET. As a control, 1 NM-IVF (ICSI) cycle with fresh ET was used. The primary outcome accounted was the livebirth rate (LBRs) following one ET; the secondary outcome was clinical pregnancy rates (CPRs), miscarriage and cycle cancellation rates. Comparisons were held over mean numbers by t-test, over median by Mann-Whitney, and categorical data were treated by Chi-square.

**Results:**

The prognosis for livebirth in the study (n=125) and control (n=208) group was equally poor (mean age: 40.2 ± 3.0 vs 40.0 ± 3.3; median AMH: 2.1 vs 2.2 (pmol/L), AFC 4.0 vs 4.0). The LBR was significantly higher with the study protocol (30.6% vs 13.3%; p=0.002), particularly in women aged 35-39 years (31% vs 10.8%; p=0.05) and 40-44 years (26% vs 10.3%; p=0.02). Lower LBR in women aged ≥35 years in the control-group was mainly attributable to the higher miscarriage rate. With significantly more oocytes (mean: 6.5 ± 3.8 vs 2.0 ± 1.4; p <0.0001) and embryos available (mean: 3.6 ± 2.3 vs 0.9 ± 0.7; p<0.0001), only a minority ended up with no ET in the study-group (7.2% vs 35.6%; p<0.0001). None dropped-out while undergoing 3 cycles, whereas no patient opted for further attempts after one standalone cycle.

**Conclusion:**

Accumulation of embryos through 3 NM-IVF cycles before transfer improves livebirth rates and reduces the risk of lacking an embryo for transfer in poor responders aged ≥35 years.

## Introduction

Optimising the outcome of in-vitro fertilisation/intra-cytoplasmic sperm injection (IVF/ICSI) in women with advanced age or poor ovarian reserve (POR) has always been a challenge. Many strategies have been proposed to improve the treatment outcomes of this category of patients; however, no single intervention or modification of the treatment protocol has been proved to be clearly beneficial (Giovanale et al., 2015; Jin et al., 2018; Vaiarelli et al., 2018). Attempts to intensify ovarian stimulation for poor responders often fail to increase the number of oocytes or embryos (Bastu et al., 2016; Song et al., 2016); milder stimulation protocols have been shown to yield a similar number of good quality embryos as with high-dose regimens (Lainas et al., 2015; Revelli et al., 2014; Youssef et al., 2017), with comparable clinical pregnancy rates (CPRs) or livebirth rates (LBRs) (Ragni et al., 2012; Mohsen and El Din, 2013; Revelli et al., 2014; Bastu et al., 2016; Song et al., 2016; Nargund et al., 2017; Youssef et al., 2017). Natural/natural modified (N/NM)-IVF has also been used in women with POR. Indeed, randomised controlled trials (RCTs) comparing the outcomes between natural IVF and ‘micro-dose flare’ (Morgia et al., 2004) or high-dose antagonist protocol (Kim et al., 2009) were found N/NM-IVF to be equally effective, in terms of CPR as well as LBR. A more recent retrospective study on poor responders reported significantly higher LBRs with NM-IVF compared to conventional antagonist cycles (Lainas et al., 2015). The perceived advantage of obtaining comparable success with less medication, better tolerance (de Klerk et al., 2007; Hojgaard et al., 2001) and a lower cost (Ragni et al., 2012) led the Practice Committee of American Society of Reproduction Medicine recommend mild approach IVF in treating poor responders (Practice Committee of the American Society for Reproductive Medicine, 2018).

Disappointing outcomes in the treatment of women with POR or advanced age with a single IVF/ICSI cycle is usually due to high cycle cancellation rates or unavailability of good quality embryos for transfer. As a result, attention has been focused on the cumulative success from multiple IVF/ICSI cycles (Nargund et al., 2001; Ubaldi et al., 2010). Freezing of oocytes in consecutive stimulated cycles with the intention of increasing the number of embryos, before contemplating ET, has emerged as a promising strategy with significantly improved pregnancy outcomes (Cobo et al., 2012; Greco et al., 2013; Ubaldi et al., 2010).

In this manuscript, we describe the first study to determine the clinical outcomes of transferring vitrified-thawed embryos from a pool of embryos accumulated over 3 consecutive NM cycles. For women with very low ovarian reserve where the chance of obtaining an embryo is uncertain, freezing embryos rather than oocytes appeared to be a more sensible option to the clinicians as well as for the patients. Thus, we hypothesized that the accumulation of frozen embryos over 3 consecutive IVF(ICSI) cycles would provide a greater number of good quality embryos for transfer and improve LBRs. To eliminate the bias incurring as a result of multiple attempts of ETs in the study group influencing the result, the LBRs following 1 ET of this ‘triple freeze programme’ were compared with those of a single NM-IVF/ICSI cycle and 1 fresh ET.

## Methods

### Ethical approval

This study did not require ethical approval.

### Study design

All consecutive NM-IVF(ICSI) cycles on women who met the Bologna criteria of POR (stated below) were included. The study was performed retrospectively, covering the period from January 2014 to December 2015. Those patients who had frozen transfers extending beyond the stipulated study period were excluded. All couples meeting our eligibility criteria were offered a ‘package’ of 3 IVF(ICSI) on NM protocol with all transferable embryos electively vitrified until the 3rd oocyte retrieval (OR), followed by transfer of one or more embryos until successful pregnancy or all embryos were utilized. Those couples who opted out of this ‘study protocol’ and intended to undergo a single NM cycle with fresh ET constituted the ’control group’. Therefore, the selection of the treatment regimen was solely based on patient’s choice, before initiation of their very first IVF(ICSI) treatment in our centre. No patients dropped out before completion of the intended treatment plan in either group. 

### Participants

The NM-IVF(ICSI) protocol was applied to all women fulfilling 2 of the 3 Bologna criteria for POR i.e. women’s age >40 years; Anti-Mullerian Hormone (AMH) <7 pmol/l, and or <7 antral follicle count (AFC), and previous poor response with conventional IVF(ICSI) (Ferraretti et al., 2011). Upper age limit for women was 44 completed years. Elective oocyte/ embryo freezing cycles, donor’s oocyte cycles or where NM cycles were undertaken to avoid ovarian hyperstimulation syndrome were excluded. All women were counselled for low chance of success and the alternative option of using donor’s oocyte was offered, quoting estimated success rates for each option.

### Protocol

All treatment cycles were started with women’s spontaneous menstrual cycles and were monitored by trans-vaginal ultrasound scans and serum estradiol (E2) and luteinising hormone (LH) from day 5 and continued every other day until the decision to trigger ovulation. GnRH-antagonist, cetrorelix acetate (Cetrotide® Merck) 0.25 mg/day was administered to suppress premature LH surge once the leading follicle(s) reached 12-14 mm and serum E2 levels exceeded 500 pmol/l. Gonadotropin (Gn) add-back was provided with a fixed daily dose (150 IU) of either recombinant follicle stimulation hormone (r-FSH) (Gonal-f®; Merck), or rarely with human menopausal gonadotropin (hMG) (Menopur, Ferring Pharmaceuticals). Final oocyte maturation (ovulation ‘trigger’) was achieved by injecting 500 mcg of chorio-gonadotropin- alfa-, (ovitrelle®; Merck) when the leading follicle(s) achieved a mean diameter of 16 mm or more with a satisfactory E2 level (>650 pmol/l). In a small number of of cycles, tamoxifen was administered until ovulation trigger to block the LH surge for women who did not want antagonist injections. Some women had hCG trigger only on their natural cycles. Selection of these three types of ‘natural modified’ protocol was based on the response of previous cycle (poor follicular development and or poor quality of oocyte) or on the patient’s choice. Occasionally, Indomethacin was added if the risk of premature ovulation was perceived (e.g. high serum LH levels). OR was carried out 35 hours after the ovulation trigger. Insemination by IVF or ICSI was performed according to pre-determined clinic policy.

For women within the Study Group, the created embryos were electively vitrified on Day 2 or 3 in the 1st, 2nd and 3rd treatment cycles, with the option of fresh ET in the 3rd cycle. The treatment cycles were in 3 successive cycles, with no gap in-between. Subsequently, frozen-thawed embryo(s) were transferred on a natural cycle with progesterone luteal phase support as described below. In principle, usually 2 (occasionally 3, if the couple opted for due to low-grade of the embryos or advanced age, after being counselled for the risk of multiple pregnancy) embryos at cleavage stage (rarely blastocyst) were transferred unless only 1 was available or there was other specific indication(s). Patients who opted for one standalone cycle (Control Group) had all fresh ETs and, if required, subsequent vitrified-thawed surplus embryo transfer following the same transfer policy. Patients in the Study Group mostly had vitrified-thawed embryo transferred (61.6%), less often fresh (24.8%) or mixed fresh and frozen (13.6%) embryos were transferred if the patient wanted, only in the 3rd and final cycle. Luteal phase support was given in the form of vaginal progesterone pessaries (cyclogest 400 mg twice daily or uterogestan 200 mg thrice daily which was continued until the pregnancy test was performed by estimating serum beta-HCG. Clinical pregnancy was confirmed by the presence of fetal heartbeat on TVS, 4 weeks after OR and the luteal support was extended till 12 weeks of gestation. Any remaining suitable embryo or embryos were cryo-preserved for subsequent transfer(s).

### Measured outcomes

LBR per single ET was measured as a primary outcome. Secondary outcomes included: mean number of retrieved oocytes and embryos created, cycle cancellations, miscarriage and multiple pregnancy rates. 

### Statistics

Normally distributed continuous variables were expressed by mean ±2 standard deviation (SD) and compared by unpaired t-test. Variables with skewed distribution were expressed by median (range) and compared by Mann-Whitney test. The categorical data were presented as number (percentage) and compared by chi-square or Fisher’s exact test. P<0.05 was considered statistically significant. Stats Direct software was used for all statistical analysis (StasDirect version 2.8.0 (27th October 2013).

## Results

**Figure 1:**
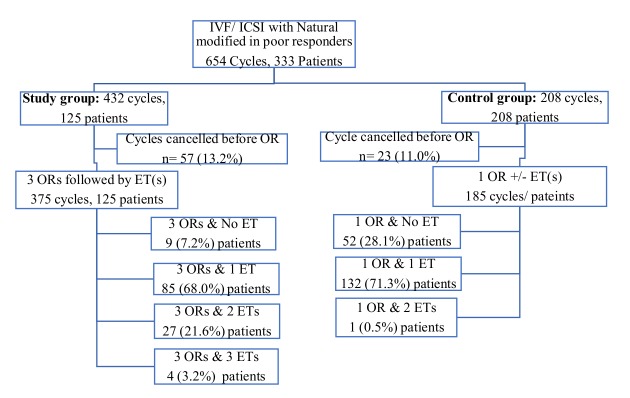
Flow chart

The distribution of couples in the case and control groups through the course of treatment is shown in the Flow Chart (Figure 1). A total of 432 cycles were started, with 125 patients in the study group and 208 patients in the control group.

No difference was observed in the personal characteristics including age, body mass index (BMI), AMH, AFC or type of infertility (primary or secondary) between the groups (Table I). The characteristics of the IVF(ICSI) cycles including the proportion of ICSI cycles and the variations in the NM protocols were no different between the study and control groups (Table I). The vast majority of the cycles were conducted on the NM protocol using GnRH-antagonist with low-dose Gn addback. Extended course of tamoxifen until ovulation trigger (without antagonist) and unstimulated cycle with only hCG trigger were employed in a minority of cases. These small variations in the NM protocol were similarly distributed between cases and controls (Table I).

**Table I:** — Personal and cycle characteristics.

	Study group n= 125 patient	Control n= 208 patients	p
Age: mean (±2SD) years ^1^	40.2±3.0	40.0±3.3	NS
BMI: mean (±2SD) kg/m^2^ ^1^	25.0±5.5	24.0±4.5	NS
AMH: median (range) pmol/l ^1^	2.1 (0.2-25)	2.2 (0.2-22)	NS
AFC: median (range) ^1^	4.0 (1-13)	4.0 (1-18)	NS
Secondary infertility: %(n) ^1^	12.0% (15)	12.0% (25)	NS
Cycle characteristics Natural modified protocols: %(n) ^2^			
- antagonist add-back low-dos Gn	88.5% (332)	88.9% (185)	NS
- hCG trigger only	6.7% (25)	8.6% (18)	NS
- Tamoxifen and Gn	5.1% (19)	2.4% (5)	NS
ICSI: %(n) ^2^	67.5% (253)	61.5% (128)	NS
Cancelled before OR: %(n) ^2^	13.2% (57)	11.0% (23)	NS
Started cycle with no embryo for ET: %(n) ^2^	37.0% (160)	36.0% (75)	NS
Single embryo transfer: %(n) ^3^	27.8% (42)	61.6% (85)	<0.0001
Double embryo transfer: %(n) ^3^	58.9% (89)	36.8% (50)	<0.0001
Triple embryo transfer: %(n) ^3^	7.9% (12)	1.2% (3)	<0.0001
Fresh ET	24.8% (31)	98.6% (205) *	
Vitrified-thawed ET	61.6% (77)	1.9% (4)	
Mixed fresh and vitrified-thawed ET	13.6% (17)	0	

1= per patient, 2= of all cycles, 3= of all ET. BMI= body mass index, AMH= Anti-mullerian hormone, AFC= antral follicle count, Gn= gonadotropin, OR= oocyte retrieval, ET= embryo transfer, NS= not significant.

*1 patient had fresh followed by vitrified-thawed ET.

There was no significant difference in the cycle cancellations before OR (Table I). However, only 7.2% of women in the ‘3-cycles group’ ended up with no embryo for transfer; while 35.6% patients in the single cycle group failed to reach the stage of ET following OR (p<0.0001). Among women who had ET in the latter group, only one had embryos available for more than one transfer procedure; while 21.6% and 3.2% women in the study group had a second and third attempt of ET respectively (Figure 1). The study group allowed a significantly higher proportion of transfer procedure with multiple embryos (usually 2 embryos), while most women in the single cycle group had just one embryo available for transfer (Table I). A majority of the ETs were undertaken in cleavage stage day 2 in both study (74.1%) and control (87.7%) populations; only 2 ETs in either the group were at blastocyst stage. Most patients (61.6%) in the study group had vitrified-thawed ET or a mixed fresh-frozen ET (13.6%), while 98.6% women had fresh ET in the control group. Suboptimal endometrial thickness or presence of polyps were the reasons of not having fresh ET in the control group. Furthermore, the mean number of retrieved oocytes and embryos created were significantly higher in women within the Study Group compared to those in single cycle group (Table 2). This resulted in significantly more patients in the Study Group who had had 2, 3 (43.2%) or ≥ 4 (40.8%) available embryos at the initiation of ET (Table II).

**Table II:** — Comparison of secondary outcomes.

Outcomes	Study group n=125	Control n=208	p
Available oocytes (mean ±2SD) ^1^	6.5±3.8	2.0±1.4	<0.0001
Available embryo @ ET (mean ±2SD) ^1^	3.6±2.3	0.9±0.7	<0.0001
No embryo for transfer^1^	7.2% (9)	35.6% (74)	<0.0001
Single available embryo^1^	8.8% (11)	38.0% (79)	<0.0001
2-3 available embryos^1^	43.2% (54)	21.6% (45)	<0.0001
≥4 embryos available for transfer^1^	40.8% (51)	0.5% (1)	<0.0001
Multiple pregnancy (twin)^1^	2.4% (3)	1.0% (2)	NS

On the primary comparison between 3 ORs followed by 1 ET in the study group and 1 OR with 1 ET in the control, the LBR was significantly higher in the former (30.6% vs 13.3%; p= 0.002). The CPRs were not significantly different in any of the age groups and the higher LBR in the 35-39 and 40-44 age groups was mainly due to a high miscarriage rates in the older age groups (Table III).

**Table III:** — Comparison of pregnancy (primary) outcomes- over all and in different age-groups.

Age groups		Study group %(n)	Control %(n)	p
Pregnancy outcome with 1 ET per patient:				
All age:	CPR/ patient	36.5% (31/85)	25.2% (34/135)	0.05
	LBR/ patient	30.6% (26/85)	13.3% (18/135)	0.002
	Miscarriage rate/ patient	10.6% (9/85)	18.5% (25/135)	NS
< 35 years:	CPR	57.1% (4/7)	54.5% (6/11)	NS
	LBR	57.1% (4/7)	45.4% (5/11)	NS
35 - 39.9 years:	CPR	34.5% (10/29)	24.3% (9/37)	NS
	LBR	31.0% (09/29)	10.8% (4/37)	0.05
40 – 44.9 years:	CPR	34.0% (17/50)	20.7% (18/87)	NS
	LBR	26.0% (13/50)	10.3% (9/87)	0.02
Cumulative pregnancy per patient (all age)			
	CPR	30.9% (39)	17.3% (36)	0.004
	LBR	23.0% (29)	8.6% (18)	0.0003

ET= embryo transfer, CPR= clinical pregnancy rate, LBR= livebirth rate, NS= not significant

Across all age groups, cumulative ET’s resulted in higher CPR and LBR’s per patient in the study group (Table III); however, the livebirths from the transfers additional to the single transfer resulted in only 10% rise in the LBR.

## Discussion

Patients included in our study were poor responders, with the median AMH in the study and control groups being 2.1 and 2.2 pmol/l, respectively. Nearly 2/3rds of the patients included (n=135) being between 40 and 44 years old, our study is one of the few to deal with such a high proportion of older women undergoing IVF/ ICSI with their own eggs. Also, this is a first study to report the outcomes of repeated embryo freezing instead of oocyte freezing.

Despite most of the couples had been recommended to have donor’s egg before joining our programme, in this study we demonstrated an improved treatment outcome with the policy of accumulating embryos through three consecutive NM-IVF cycles prior to ET within a poor prognosis population. To eliminate bias incurring from more attempts of ET from accumulated embryos in the study population (multiple ET procedures was possible for 1 in 4 women in the Study Group, while 1 woman in the control), our primary analysis of livebirth outcome was based on women who had only 1 ET procedure in both study and control groups. An ET undertaken from the pooled embryos resulted in significantly higher LBR when compared with a fresh ET following a single cycle (Table III). However, the benefit was only found in older women between 35 and 39 years (LBR: 31.0% vs 10.8%, p=0.05) and 40 and 44 years age groups (LBR: 26.0% vs 10.3%; p=0.02). Even in these age groups, apparently higher CPRs failed to achieve statistical significance. Significantly lower LBRs in the control groups was mainly due to the higher miscarriage rates (Table III). Importantly, these findings support our original hypothesis that a better selection of good quality oocytes and hence embryo(s), from a larger cohort, might have resulted in an improved outcome. Over 43.0% of patients in included in study arm had >1 embryo available for transfer, compared to only 21.6% in the control group (p<0.0001) which made double (rarely triple) embryo transfer more often possible (Jonsdottir et al., 2011). The enhanced success in our study population could be due to higher chances of transferring an euploid embryo from a larger cohort (Chamayou et al., 2017), given that aneuploidy remains a rate-limiting factor, particularly in older women undergoing IVF(ICSI) treatment. Future studies may explore this hypothesis further. We believe that the difference in the embryo transfer protocols (Natural cycle frozen-thawed versus Natural-modified cycles) were unlikely to have a significant impact on implantation rate. Indeed, fresh transfers were also subsequent to a ‘natural’ IVF cycles where a small dose of gonadotropin was administered as an ‘addback’ therapy along with GnRH-ant to prevent premature LH surge.

Additionally, the per patient cumulative CPRs and LBRs including all ETs was also significantly higher with this study protocol (23.2%) than that of patients in the matched control group who had single NM cycle followed by ET (8.6%, p= 0.0003). Another important factor that led to better per patient success in the study population was that only a few poor responders (7.2%) in the study group were left with no embryo available for transfer whereas around 1 in 3 women had so in those in the control group (p<0.0001). Also, no significant differences in CPRs or LBRs were observed between the study and control groups in women <35 years which may reflect an age-related better quality of embryos obtained from NM cycles, despite an unexpected decline in the ovarian reserve. Whether low AMH levels in younger women is associated with high rate of embryo aneuploidy is a matter of controversy; in this line, recent studies have reported a comparable ploidy status, blastocyst formation and LBRs in younger women with normal and diminished ovarian reserve (Jiang et al., 2018; Morin et al., 2018).

The concept of accumulation of oocytes through repeated IVF cycles was first published by Cobo et al. (2012). In this report oocytes were retrieved through multiple conventional antagonist cycles and vitrified until at least 5 embryos were anticipated to be obtained for subsequent ET(s). This prospective trial on 482 poor responders found significantly higher cumulative LBRs/patients compared to those who had single cycle with fresh ET (36.4% vs 23.7%). In women of >40 years, the LBRs of 15.8% upon oocyte vitrification and 7.1% in fresh ET group were very similar to those reported here. Subsequently, Greco et al. (2013) applied the same principle in a retrospective study, but with a NM-IVF protocol and in a smaller cohort (n=67) of patients with POR. They reported a significantly higher CPR with SET from accumulated oocytes over 3 vitrified-thawed cycles plus 1 fresh cycle, when compared with up to 3 repetitive fresh cycles with SET. Again, the CPR (34.4%) with accumulated oocyte group in this study was close to our findings reported here. In contrast to these two studies that electively vitrified oocytes, we cryopreserved embryos. The mean age of women in our study was greater than the mean of either of these two studies. We drew an upper limit of 3 NM-cycles, regardless of the number of embryos obtained. As such, our study is the first to present retrospective data on elective embryo freezing rather than oocyte freezing. Similar to our study, Greco et al. (2013) selected a NM-IVF protocol in preference to conventional high-dose protocols for women with POR, in order to make repeated IVF cycles more ‘patient friendly’ and thus to prevent women from dropping-out of treatment.

Consistently, good cumulative outcomes have been associated to N/NM-IVF (Nargund et al., 2001; Morgia et al., 2004; Pelinck et al., 2007). It has also been associated with lower treatment cost, particularly for poor responders undergoing multiple cycles (Morgia et al., 2004; Reyftmann et al., 2007; Groen et al., 2013). Moreover, N/NM-IVF has been linked with better endometrial receptivity (Reyftmann et al., 2007; Verberg et al., 2008) with comparable, if not better implantation rates and LBRs in women with POR (Morgia et al., 2004; Papaleo et al., 2006; Matsuura et al., 2008; Lainas et al., 2015).

In two publications, Pelinck et al. (2006, 2007) identified acceptable cumulative PR after 3 isolated full N-IVF cycles which continued to increase up to 9 cycles; however, the drop-out rates also tended to rise when the treatment extended towards 9 cycles. For this reason, we adopted a policy of 3 cycles which was a “trade-off” between success and patients’ compliance, and possibly cost. In our study, none of our patients who opted for 3 cycles of ORs followed by ET dropped out before treatment completion. It can be argued that cumulative success of up to 3 full cycles with fresh ET(s) might have been as good as that found in our study group, with the obvious advantage of avoiding unnecessary further treatment cycles, in case livebirth is achieved in the first or second cycle. Needless to mention, very few patients might have had the mental strength to pursue treatment up to 3 cycles after one or more failed cycle(s), despite this being offered as a ‘package’. In our observation, completion of 3 cycles before ET reduced the apprehension of cycle cancellation or the stress of waiting for the outcome after each ET in single cycles (with less chance of having surplus embryos); indeed, all those in the control arm who had a failed single cycle did not opt for a further cycle. It can also be argued that multiple ORs and cryo-preservation of embryos might have incurred in a higher cost compared to that with a single cycle. This strategy subjected women to repeated invasive OR procedures. Nevertheless, the strikingly higher success with the 3-cycle accumulation strategy, coupled with the less stress associated with the natural modified protocol could justify our strategy, outweighing the perceived risks. Of note, no major incident was reported from a total of 644 retrieval procedures performed during the study period, with zero drop-out incidence. Plus, the cycles were conducted back-to back, in consecutive menstrual cycles, minimizing the length of the entire treatment course. We must highlight that repeated failed attempts with full cycle could be an expensive option for POR patients with a higher chance of ending up with no embryos for transfer and a predicted livebirth of only <10%. Indeed, most of the couples who opted for 1 attempt at a time could not afford for further cycles. Estimation of overall cost, including that from pregnancy and childbirth (taking multiple pregnancies into account) is possible in a pre-planned prospective trial, therefore, economic evaluation was not sought for in our retrospective analysis. The incidence of multiple pregnancy was unlikely to have a major impact on the overall cost, as the incidence was very low among the poor responders (2.4% in the study group and 1.0% in the control group) and not significantly different, regardless of the relative difference in the number of embryos transferred. Whether our three-cycles of freezing strategy is a cost-effective option in countries where a robust reimbursement policy is in place needs careful evaluation through a health-economic model in a prospective trial.

Further, we have minimized the chance of selection bias of this retrospective study by rigidly adhering to the inclusion and exclusion criteria. Personal and cycle characteristics including the stage of embryos at the time of transfer were similar among the cases and controls. Also, questions may be raised in regards of pregnancy outcomes presented in this study within NM cycles has not been ‘tested’ against common contemporary practice which usually constitutes of one of the high-dose conventional IVF regimens targeting a maximum yield of oocytes or embryos. However, to this date, published data does not suggest any better pregnancy or delivery rates with conventional high-dose cycles, when compared with those of NM cycles in poor responders, despite harvesting higher number of oocytes with the former (Kim et al., 2009; Lainas et al., 2015). With the emerging evidence of equivalent or better pregnancy outcomes with frozen-thawed ET, our comparison between fresh and frozen/thaw transfer could be a matter of criticism. However, NM cycle being not dissimilar to a natural menstrual cycle, the deleterious effect of supra-physiological level of reproductive hormones possibly could not be applied with our treatment protocol. Published data in the literature do support this theory (Celik et al., 2015; Xue et al., 2018).

In closure, the message from this study resolves in that an accumulation of embryos through multiple cycles might give the opportunity of better selection and transfer of multiple embryos, leading to improved pregnancy outcomes in poor prognosis women over the age of 35 years. In contrast to single cycle with high risk of cycle cancellation, the vast majority of women with three repeated cycles would have at least 1 embryo for transfer. Working on this principle, accumulation of enough embryos, to expect at least one euploid embryo, through a PGS program could be considered to be very effective (and cost-effective) for older (>35 years old) women with diminished ovarian reserve seeking for IVF/ICSI treatment with their own oocytes.

## Funding:

No funding was required to conduct this study.
